# The Eye as a Window to Systemic Infectious Diseases: Old Enemies, New Imaging

**DOI:** 10.3390/jcm8091392

**Published:** 2019-09-05

**Authors:** Vittorio Pirani, Paolo Pelliccioni, Serena De Turris, Alessandro Rosati, Alessandro Franceschi, Claudia Cesari, Michele Nicolai, Cesare Mariotti

**Affiliations:** Eye Clinic, Polytechnic University of Marche, 60126 Ancona, Italy (V.P.) (S.D.T) (A.R.) (A.F.) (C.C.) (M.N.) (C.M.)

**Keywords:** infectious uveitis, syphilis, tuberculosis, toxoplasmosis, fluorescein angiography, indocyanine green angiography, optical coherence tomography, optical coherence tomography angiography, fundus autofluorescence

## Abstract

Background: Syphilis, tuberculosis and toxoplasmosis are major infectious diseases worldwide; all of them are multisystem pathologies and share a possible ocular involvement. In this context, a fundamental help for the definitive diagnosis is provided by the ophthalmologist, through clinical evaluation and with the aid of a multimodal imaging examination. Methods: We hereby describe selected cases who came to our attention and were visited in our eye clinic. In all clinics, the use of retinal and optic disc multimodal imaging during ophthalmological evaluation allowed to make a diagnosis of an infectious disease. Results: In our tertiary referral center more than 60 patients with syphilis, tuberculosis and toxoplasmosis have been evaluated in the last two years: In 60% of cases the ophthalmological evaluation was secondary to a previous diagnosis of an infectious disease, while in the remaining cases the ophthalmologist, with the help of a multimodal imaging examination and clinical evaluation, represented the physician who leads to the diagnosis. Conclusion: Our results confirm how in these life-threatening pathologies a prompt diagnosis is mandatory and may benefit from a multidisciplinary and multimodal imaging approach, especially during ophthalmological evaluation.

## 1. Introduction

Human population has always been facing infectious pathogens, a universal enemy of all living organisms. Whether caused by viruses, parasites or bacteria, infectious diseases have been and will be one of the most feared and deadly causes of mortality. A milestone in this never-ending struggle has been reached in very recent times, due to antibiotic therapy, completely revolutionizing the approach of humanity and medicine to infections.

In this study, our attention will focus on three pathogens: The bacteria *Treponema pallidum* and *Mycobacterium tuberculosis*, and *Toxoplasma gondii*, an intracellular parasite belonging to the *Eukaryota* domain.

Syphilis, caused by *Treponema pallidum*, is a common infective pathology worldwide with an estimated 10–12 million new infections each year [1,2]. However, there has been a recent resurfacing of this pathology, especially among men who have sex with men and drug users, as a coinfection with human immunodeficiency virus (HIV) [3]. Syphilis has a complex and variable clinical presentation and history, as it can affect different organ systems (including skin, heart, blood vessels, bones, nervous system and the eye) and as it embraces three progressive clinical stages with chronological overlap [4]. The same ocular syphilis can also show multiple ways to manifest itself. Therefore, it is no wonder that this infection is also known as ‘the great imitator’ for its ability to mimic many diseases, due to its extensive range of possible clinical manifestations [5]. Ocular referred symptoms can be in fact eye pain, vision loss, floaters, flashing lights or photophobia. Almost every structure of the eye can be affected, and clinical presentation include syphilitic conjunctivitis, interstitial keratitis, scleritis, chorioretinitis with vitritis, retinal vasculitis, neuroretinitis with optic disc involvement and exudative retinal detachment, both in the early and late stages of the disease, in patients with or without HIV coinfection [6,7].

Tuberculosis represents the leading cause of death from an infectious disease with an estimated 10.4 million new cases in 2015, of which 10% were among children and 12% involved HIV coinfection [8]. The global burden of *M. tuberculosis* infection has recently been re-estimated at 24% [9,10]. For these reasons, tuberculosis is a global health problem. Tuberculosis affects the lungs in 80% of patients, with other organs involvement (including the eye) in the other 20% of cases. It is mandatory for physicians to think of this diagnosis during clinical evaluation, as ocular involvement can manifest itself in a similar way to that of more common pathologies causing ocular inflammation of the different eye segments. The most common form of ocular involvement derives from hematogenous spread, therefore, the uveal tract (i.e., iris, ciliary body, and choroid) is frequently involved because of its high vascular content [11]. Choroidal lesions, such as focal, multifocal, or serpiginous-like choroiditis can be seen, and also retinal lesions, such as retinal vasculitis. Other relevant ocular complications are optic nerve lesions, like optic disc granuloma or optic neuritis, and intermediate or anterior uveitis [12,13].

Toxoplasmosis is one of the most common human parasitic zoonoses, infecting approximately 25–30% of the world population [14,15]. However, seroprevalence varies widely, from 10 to 80% between and within countries and geographic regions [16], from a minimum prevalence in Southeast Asia, North America and Northern Europe to a maximum prevalence in Latin America and in tropical African countries. Recurring posterior uveitis is a common clinical presentation of this disease, often associated with unilateral, necrotizing retinitis and secondary choroiditis, arising adjacent to a pigmented retinochoroidal scar, and also with retinal vasculitis and vitritis. Multiple unusual manifestations are documented, and severe inflammation is observed in patients with immunodeficiency [17].

All three of these infectious diseases are systemic pathologies that share a possible ocular involvement. In this context, a fundamental help for the definitive diagnosis is provided by the ophthalmologist. In fact, there are many cases in which the patient, in the early stages of the disease, may still be asymptomatic except for visual disturbances; in these cases the ophthalmologist may play a key role, being the first figure in the clinical pathway and the physician who leads to a diagnosis of infectious disease. Consequently, the eye becomes a window through which to look at the whole organism.

We hereby describe selected cases who came to our attention and were visited in our eye clinic. In all clinics, the use of retinal and optic disc multimodal imaging during ophthalmological evaluation allowed to make a diagnosis of an infectious disease.

We have chosen to focus our attention on these three worldwide infections, and to summarize the major ocular clinical presentations.

## 2. Experimental Section

*Methods*

This was an original case series with a literature review that adhered to the tenets of the Declaration of Helsinki. It adhered to the 1964 Helsinki declaration and its later amendments. Informed consent was obtained from all individual participants included in this paper, and it was approved by the Local Institutional Review Board (IRB).

### 2.1. Syphilis

**Figure 1:**
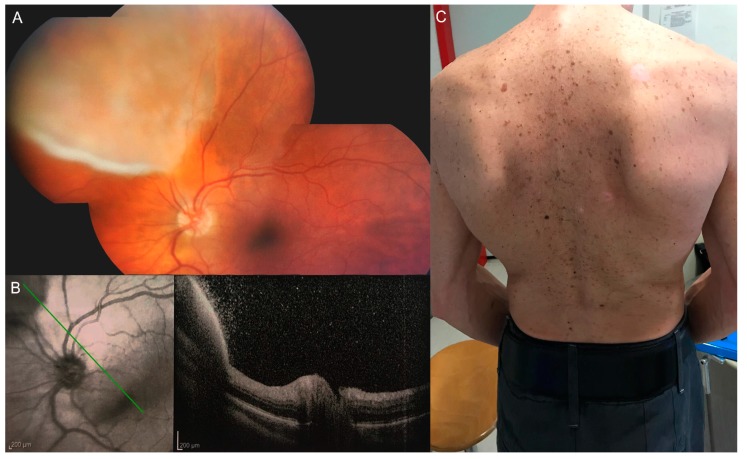
Presentation of syphilis. Fundus examination (**A**) shows the presence of vitreous inflammation and multiple superficial pre-retinal aggregates are evidenced on optical coherence tomography (OCT) scans (**B**). The patient presented a form of tertiary syphilis affecting both the eye (**A**,**B**) and the skin (**C**).

Eye involvement is very frequent during the secondary and tertiary stage of syphilis, but it has been described in all the stages of the disease [18] (Figure 1). Chorioretinitis is the most common ocular inflammation in HIV-negative subjects and occurs in 75% of the cases [19,20]; panuveitis is the most common manifestation in HIV-positive patients [21]. Several reports described an increased number of HIV-positive patients with ocular syphilis in the last years [3], suggesting that these patients may often present ocular syphilis before HIV infection is diagnosed [21]. Moreover, HIV co-infection in patients affected by syphilis may modify the natural course of treponemal infection, increasing the propensity of the disease to progress to neurosyphilis [22].

Syphilitic chorioretinitis (Figure 1) is typically bilateral with small lesions in the early stages, with a tendency to converge [23]. Vascular involvement could be present and affect both capillaries and veins [24]. Vascular tortuosity, exudation, fibrosis or obstructive forms of vasculitis have been described [25] in many cases of treponemal infection. Serous retinal detachment has been reported in some patients [26]. Vascular involvement and the granulomatous nature of anterior uveitis must lead the clinician to evaluate a differential diagnosis of tuberculosis.

Clinical signs and complications of ocular syphilis are summarized in Table 1 and Table 2, as reported by Pratas et al. [27] and Zhang et al. [28].

Acute syphilitic posterior placoid chorioretinitis (ASPCC) is a rare ocular manifestation of secondary syphilis. First described by Gass et al. [29], it is characterized by the development of a large, deep, yellowish circular or oval placoid lesion at the posterior pole localized in the outer retinal layers (ORLs) that rarely extends beyond vascular arcades [30]. Its pathogenesis is probably linked to *Treponema pallidum* direct invasion of the choriocapillaris with consequent vascular obstruction or deposition of soluble immune complexes [29].

**Figure 2:**
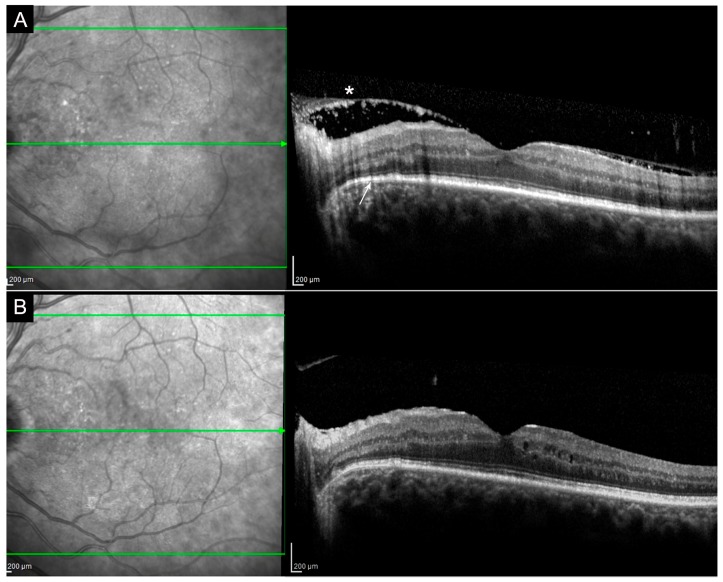
Follow-up OCT scans (**A**) showing multiple superficial pre-retinal aggregates (**asterisk**) and ellipsoid zone disruption (**arrow**) that solve after antibiotic therapy (**B**). Pre-retinal aggregates probably correspond to a combination of spirochetes and inflammatory cells. Ellipsoid zone disruption is probably caused by an accumulation of lipofuscin, inflammatory cells or incomplete phagocytosis of outer retinal segments.

Multiple superficial pre-retinal aggregates overlying inflammatory areas have been observed in some optical coherence tomography (OCT) scans of patients affected by ocular syphilis (Figure 2). These manifestations resolve after antibiotic therapy. Yang et al. proposed that these aggregates could correspond to a combination of spirochetes and inflammatory cells [31,32].

**Figure 3:**
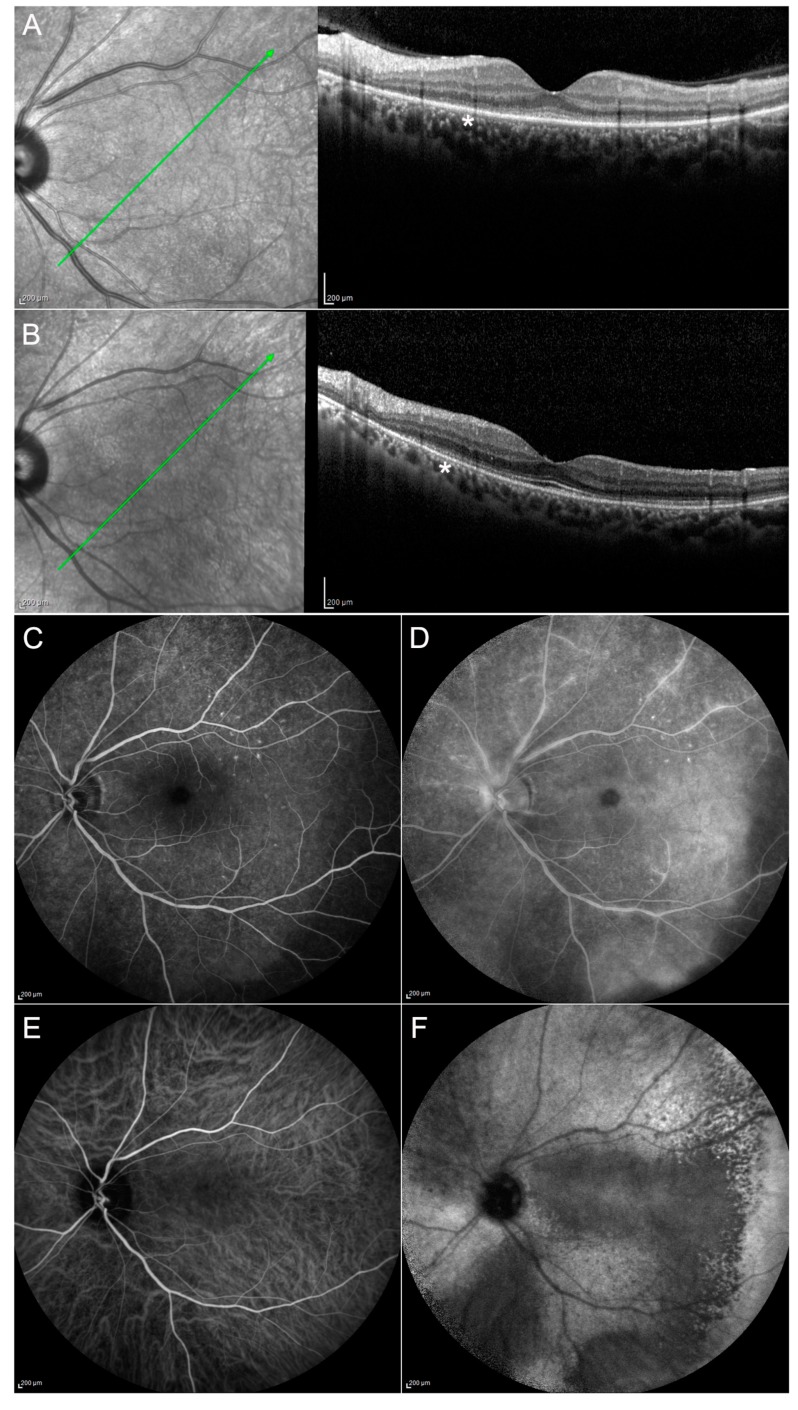
Case 1, acute syphilitic posterior placoid chorioretinitis (ASPCC). Patients affected by ASPCC show typical outer retinal abnormalities on OCT with ellipsoid zone disruption (**A**) (**asterisk**), partially resolved after the treatment (**B**). FA reveals early hypofluorescence and late hyperfluorescence corresponding to the lesions (**C**,**D**). ICGA shows hypocyanescence until the late stages of the examination (**E**,**F**). Green arrow: plane and orientation of the optical coherence tomography (OCT) line scan.

Patients affected by ASPCC show typical outer retinal abnormalities on OCT with ellipsoid zone disruption. This feature disappears after the treatment (Figure 3). These areas can be clearly observed with blue fundus autofluorescence (B-FAF) showing a hypofluorescent pattern. This is probably due to an accumulation of lipofuscin, inflammatory cells or incomplete phagocytosis of outer retinal segments [33]. Fluorescein angiography (FA) reveals early hypofluorescence and late hyperfluorescence at the posterior pole corresponding to the lesions. Indocyanine green angiography (ICGA) shows hypocyanescence until the late stages of the examination [30,34].

**CASE 1**—Clinical case presented is a 67-year-old Caucasian male, complaining about a sudden decrease of visual acuity (VA) in both eyes, weakness, dizziness and headache. He had a history of epilepsy in treatment with Valproic acid for approximately three years; he reported frequent travels in exotic countries and unprotected sexual intercourses. Best-corrected visual acuity (BCVA) was 0.70 LogMAR in the right eye and 0.60 LogMAR in the left eye. Slit lamp examination showed the presence of anterior granulomatous uveitis in both eyes with associated vitreitis. Fundus oculi was not clearly visible, due to the presence of vitreous inflammation. Small retinal hemorrhages were observed in the posterior pole along with small, diffused, circular yellowish lesions. OCT scans (Figure 3) showed the presence of superficial retinal aggregates on the vitreous hyaloid and loss of ellipsoid zone signal. FAF evidenced the presence of hyperautofluorescent areas corresponding to the retinal pigmented epithelium (RPE) irregularity observed in OCT scans. FA and ICGA showed a clinical situation characterized by the presence of vasculitis and placoid infiltrates (Figure 3).

The patient underwent treponemal and non-treponemal blood tests (TPHA, VDRL), and the results were negative. HIV serology test was performed [35], and the results were positive. The other sexually-transmitted diseases serology tests (such as viral hepatitis) were found to be negative.

**Figure 4:**
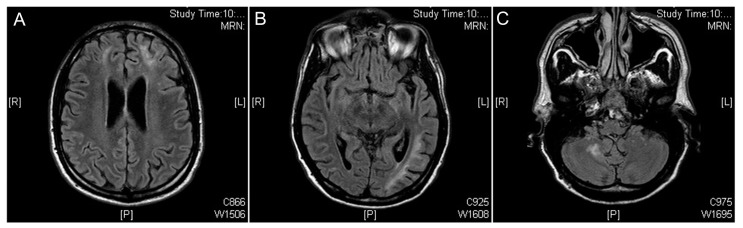
Case 1, Neurosyphilis. Magnetic resonance imaging (MRI) of the brain with gadolinium showing the presence of multiple hyperintense lesions in long repetition time (TR) sequences localized bilaterally in frontal hemispheres (**A**); in temporal, parietal and occipital left hemispheres (**B**) and in the right cerebellum peduncle (**C**). MRN: magnetic resonance neurography; R: right; L: left; P: posterior.

Magnetic resonance imaging (MRI) of the brain with gadolinium was performed (Figure 4), showing the presence of multiple hyperintense lesions in long TR sequences localized bilaterally in frontal hemispheres; moreover, in temporal, parietal and occipital left hemispheres and in the right cerebellum peduncle (Figure 4).

Because of the presence of neurological involvement, lumbar puncture was performed (in accordance to recommendations of the United States Centers for Disease Control and Prevention) [33]. Cerebrospinal fluid (CSF) examination showed high values of albumin (30.10 mg/dL; normal range: 14.00–20.00), IgG (7.2 mg/dL; normal range: 2.0–4.0); increased Link Index (0.78; normal range 0.00–0.65) and Tourtellotte Index (12.0 mg/24 h; normal range 0.0–3.0). Values of *Treponema pallidum* IgM were 1.0 using enzyme immuno-essay (EIA) method. CSF VDRL ratio was 1:4 (normal values < 1:2) and TPHA/TPPA ratio was 1:320 (normal values < 1:80). HIV1 RNA real time (RT) PCR on CSF resulted positive (72 copies for mL).

From a recent systematic review and meta-analysis, including 32 previous studies, VDRL is reported to be the most common test used to detect neurosyphilis, with a mean positivity rate of 34.8% [28].

The patient was treated with a standard regimen [36]: Intramuscular procaine penicillin G 2.4 units for 14 days and Prednisone 1 mg per kg of body weight two days after antibiotic therapy, tapered over a period of a month. Corticosteroids were used to prevent Jarisch-Herxheimer reaction, whose pathogenesis remains poorly understood [37]; it is probably caused by the release of treponemal breakout products [38]. Simultaneously, he started antiretroviral therapy (ART) for HIV infection [39]. A recent systematic review reported Jarisch-Herxheimer reaction to occur in 2.2% of treated cases [28].

After a month of follow-up, BCVA was 0.1 LogMAR in both eyes. OCT showed partial restoration of the ellipsoid zone, and there was a decrease of hyperfluorescence at B-FAF.

### 2.2. Tuberculosis

Intraocular tuberculosis is a common cause of ocular disease [40]. Multimodal imaging plays a relevant role in the detection of disease manifestations of the eye, since microbiological evidence of Mycobacterium tuberculosis is nearly always lacking in these patients.

Tuberculosis can affect all the eye structures, and the posterior pole is the most frequently involved [41]. Choroidal and retinal manifestations have been largely described in the literature. Choroidal tubercles, serpiginous-like choroiditis, Eales disease are only some examples of tuberculosis-related ocular disease [11]. Frequency of tuberculous uveitis in reported series from different countries ranges from 0.2% to 10.5% [42], and the clinical features of patients with Tubercular uveitis have been recently reported [43] and are summarized in Table 3.

**Figure 5:**
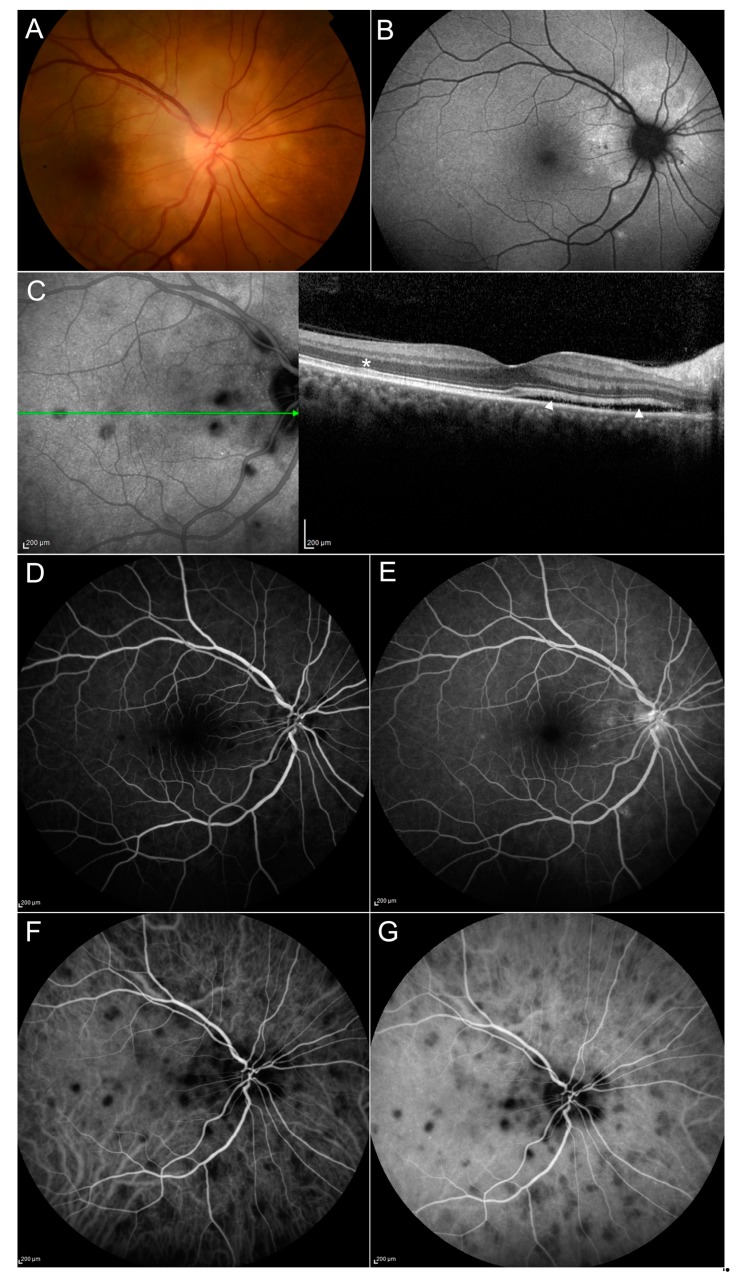
Case 2, ocular tubercles. Fundus photo (**A**) evidences the presence of multiple, yellowish lesions with indistinct borders. Edema around the optic disc (**A**) is better evidenced with B-FAF (hyperfluorescent) and OCT scan (**C**), showing the area of retinal pigmented epithelium (RPE) detachment (**arrowheads**). Contact sign over the tubercle can be seen (**asterisk**) on OCT (**C**). Granulomas are characterized by early hypo-fluorescence (**D**) and late hyper-fluorescence (**E**) after fluorescein injection. ICGA examination shows the presence of regular-shaped, hypocyanescent lesions from the early (**F**) to the late stages (**G**) of the examination.

Choroidal tubercles have been reported in many patients affected by ocular tuberculosis (Figure 5). Their size ranges from 0.5 to 3.0 mm, and the lesions are usually multiple and may appear at the fundus examination as yellow, white or gray. The most frequent localization is the posterior pole, and the lesions may be accompanied by hemorrhages, exudates or perilesional edema [44,45]. Choroidal tubercles may not be present in the early phases of the disease, and fundus changes should be checked carefully over time [46]. Mehta and al [47] found choroidal tubercles in 34.6% of 52 patients with neurotuberculosis, diagnosed by the presence of intracranial granulomas or tuberculous meningitis.

Choroidal lesions show a typical pattern at FA characterized by early hypo-fluorescence and late hyper-fluorescence after dye injection. ICGA is very useful to determine primary disorders of the choroid. Granulomas are not filled by indocyanine because of their mass effect, therefore, they appear as regular-shaped, hypocyanescent lesions from the early to the late stages of the examination [13].

OCT can show the presence of adhesions between retinal pigmented epithelium (RPE)—choriocapillaris layer and neurosensory retina over the granuloma [48]. This was defined by Salman et al. “contact sign”, and is probably due to inflammatory adhesions between RPE and neurosensory retina. Enhanced depth imaging OCT (EDI-OCT) shows hypo- or iso-reflective areas within the choroid with loss of the typical vascular pattern.

**Figure 6:**
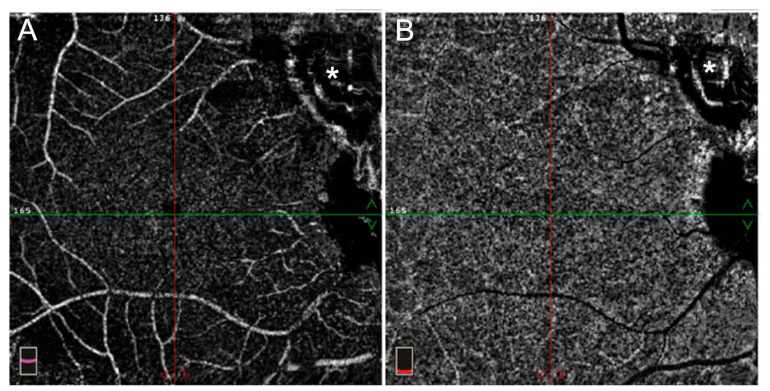
OCTA. Areas of flow void (**asterisk**) in superficial (**a**) and deep (**b**) vascular plexus, corresponding to granulomas with vessels starting to grow irregularly at the edge of the lesions during the healing process.

OCT Angiography (OCTA) can show areas of flow void (capillary hypoperfusion), corresponding to granulomas with vessels starting to grow irregularly at the edge of the lesions during the healing process [49] (Figure 6).

**Figure 7:**
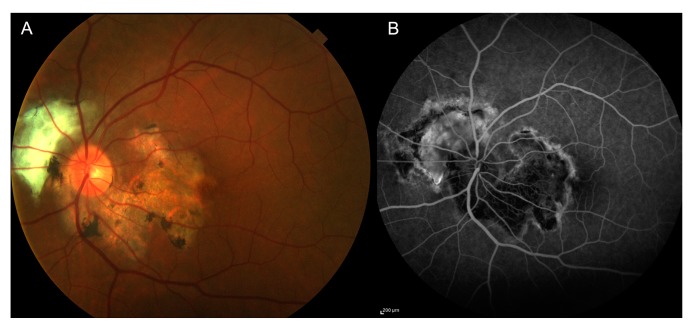
Serpiginous-like choroiditis, clinical presentation. Lesions are usually localized around the optic disc (**A**) and are characterized at FA by late hypo-fluorescence (**B**); an area of retinal atrophy is localized next to the optic disc (**A**,**B**).

Serpiginous-like choroiditis (Figure 7) usually affects choroid and is most frequently localized around the optic disc. Lesions may be solitary with a plaque-like aspect or can be multiple and diffused at the posterior pole. They can be at first isolated, then converge in the late stages of the disease [50,51]. They show a typical pattern at FA characterized by early hyper-fluorescence and late hypo-fluorescence. ICGA lesions appear hypocyanescent in all the stages of the examination.

**Figure 8:**
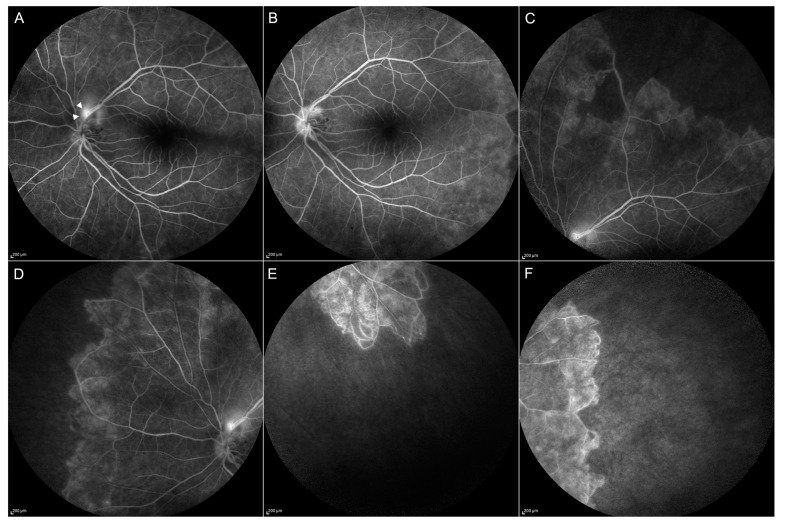
Case 3, Eales disease. Signs of vasculitis (**arrowheads**) are visible in the late phases of FA (**A**) with resolution after the therapy (**B**). Extended ischemic areas in the peripheral retina appear darker and without vascularization (**C**–**F**).

Eales disease (Figure 8) is a vaso-proliferative disorder of the retina, most common in young and healthy adults [12]. It is characterized by neovascularization, retinal ischemia and recurrent vitreous hemorrhages. Eales patients usually refer to a painless visual decrease, secondary to vitreous hemorrhage. After the vitreous clears, perivascular exudates and hemorrhages are visible at fundus examination. FA and ICGA can help to detect vascular signs of the disease, such as vasculitis, venous thrombosis, ischemia and neovascularizations, and are very useful to define the treatment of the different kinds of lesions. OCTA can allow early detection of neovascular membranes, but cannot evidence blood-retinal barrier damage or the presence of ischemia as shown by dye leakage in FA [52].

**CASE 2**—The first TBC case presented is a 56-year-old Caucasian woman, reporting a decreased visual acuity in the right eye. Best Corrected Visual Acuity was 0.4 LogMAR, and anterior segment slit lamp examination showed no abnormalities. Fundus examinations (Figure 5) revealed the presence of optic disc swelling and the presence of a yellowish area around the head of the optic nerve. OCT scans revealed the presence of a serous retinal detachment. B-FAF evidenced the presence of a hyperfluorescent area around the optic disc, corresponding to the area of the detachment. Mantoux and Quantiferon tests were both found to result positive.

We performed FA and ICGA examinations (Figure 5). Contact sign was present and corresponded to FA and ICGA features of choroidal tubercles. Computed Tomography (CT) scans of the chest revealed the presence of lung granulomas. The patient underwent a standard regimen of treatment consisting of an intensive phase treatment with isoniazid, rifampicin, pyrazinamide, ethambutol for two months and a continuation phase with isoniazid and rifampicin for four months. At last follow-up, BCVA was 0.1 LogMAR.

**CASE 3**—The second TBC case is a 44-year-old, Caucasian woman complaining of bilateral painless vision loss. BCVA was 0.1 LogMAR in the right eye and 0.4 LogMAR in the left eye. Slit lamp examination showed the presence of a hemorrhage around the optic disc. OCT showed no foveal abnormalities. FA examination evidenced the presence of dye leakage around the optic nerve. FA showed a lack of vasculature in the peripheral retina, corresponding to extensive ischemic areas (Figure 8). Mantoux and Quantiferon test results were positive. Lung CT showed lesions compatible with granulomas.

Ischemic areas of the retina were treated with Argon laser. The patient underwent systemic therapy with a standard regimen for tubercular infection. After one month of treatment, BCVA was 0.0 LogMar in both eyes, and there were no more signs of vasculitis.

In all of these cases, a differential diagnosis with other granulomatous diseases was made; the revised criteria of the International Workshop on Ocular Sarcoidosis (for the diagnosis of ocular sarcoidosis) and of the International Ocular Syphilis Study Group (for the diagnosis of ocular sarcoidosis) were applied [53,54].

### 2.3. Toxoplasmosis

*T. gondii* is the leading cause of posterior uveitis worldwide [55]. The typical presentation is unilateral vitreitis associated with choroidal lesions [56]. Vision loss is caused mainly by vitreitis, but visual damage could become permanent in case of macular scars or optic atrophy [57,58]. Reported complications and manifestations of ocular toxoplasmosis are listed in Table 4, according to Butler et al. [17].

**Figure 9:**
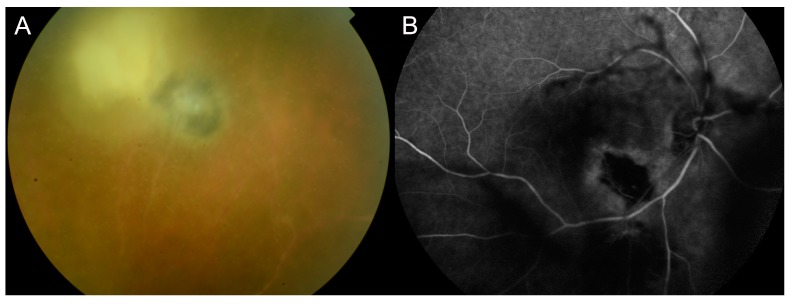
Toxoplasma gondii chorioretinitis. Severe vitreitis with “headlight in the fog” sign; a nidus of fluffy white, necrotizing retinitis or retinochoroiditis adjacent to a variably pigmented chorioretinal scar is visible at fundus examination (**A**). On FA, the lesion is hypofluorescent with the presence of a hyperfluorescent border (**B**).

The classic sign of infection consists of a nidus of fluffy white, necrotizing retinitis or retinochoroiditis adjacent to a variably pigmented chorioretinal scar [59,60]. Chorioretinitis usually involves all the retinal layers but may be limited to inner or outer retina only [17,59]. The active lesion may be obscured by severe vitreitis; this presentation is called “headlight in the fog” sign [60] (Figure 9). Anterior granulomatous or non-granulomatous uveitis can be associated with vitreitis [61], often causing an increased intraocular pressure [62]. Vasculitis can involve both veins and arteries; arteriolitis with segmental intravascular yellowish lesions (Kyrieleis plaques) has been described [63].

An atypical manifestation of the disease is punctate outer retinal toxoplasmosis. This entity is very frequent in immunocompetent young adults [59,64]. Bilateral lesions may be found in up to one-third of the patients [65]. Lesions appear as gray-white spots at the level of deep retina and RPE. Vitreous reaction may be poor or absent. Acute lesions may resolve forming fine granular white dots, approximately 25 to 75 μm in size [64]. The clinical meaning of these dots is not completely understood; they may represent focal gliotic outer retinal scars or an encysted tissue form of *T. gondii*. This clinical form of toxoplasmosis is usually located in the macular region; for this reason, visual symptoms occur early, even if the lesions are small or do not involve all the retinal layers [66].

**Figure 10:**
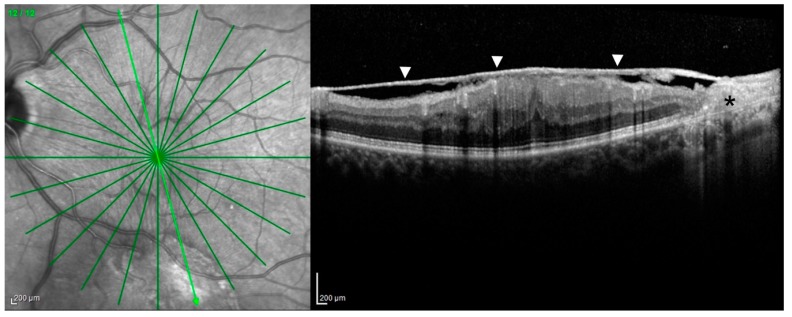
Toxoplasma gondii-related epiretinal membrane. On OCT, a hyperreflective membrane (**arrowheads**) can be seen adjacent to a toxoplasma scar (**asterisk**).

Goldenberg et al. illustrated the evolution of Toxoplasma retinal lesions in different stages of the disease, from the acute phase to resolution, using OCT [67]. In the acute phase, they described alterations of the neurosensory retina with disruption, thickening, hyper-reflectivity and photoreceptor interruption with an elevation of RPE; the choroid is thick and hyporeflective. During follow-up, choroid returns to the normal thickness and becomes gradually hyperreflective. Hyperreflective deposits can be present within the retinal interface. During the follow-up, these deposits become smaller, enter the inner retinal layers and fade with time until complete resolution. Multiple hyperreflective dots can be seen in the vitreous cavity, compatible with vitreitis. Posterior hyaloid thinning has been shown during the acute phase; in the resolution phase, posterior hyaloid detachment has been reported. Different types of scars were described [67] according to the changes occurring in the outer retina and Bruch’s membrane: Atrophic, elevated, deep and combined (atrophic and elevated). Epiretinal membranes were found on active and scarred lesions (Figure 10).

FAF signal may depend on the stage and the activity of the disease. In the early setting of the disease, a patch of reduced FAF signal with increased perilesional autofluorescence may be observed. In advanced stages, retinal scarring and atrophy develop and B-FAF signal is low. Satellite lesions may be hyper- or hypofluorescent [68].

FA and ICGA may be useful to monitor the evolution of the lesions. FA is very useful to detect vasculitis, shunts, vascular occlusion, macular edema, neovascularizations or in case of papillitis [69]. Active lesions stain gradually, starting from the borders. The presence of a serous retinal detachment appears hyperfluorescent in the late stages of the examination. After the acute stage of the disease, edema gradually resolves. FA highlights pigmentary changes of the lesions and scar formation in the resolution phase [68]. ICGA may be useful in assessing the extent of choroidal involvement and the evolution of lesions, especially during the follow-up.

**Figure 11:**
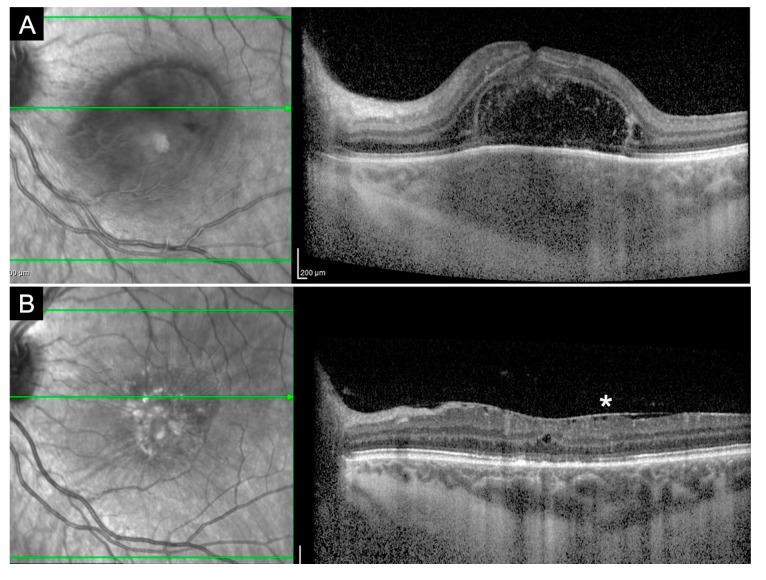
Case 4, Punctate outer retinal toxoplasmosis. OCT in the acute phase (**A**) showing alterations of the neurosensory retina. Hyperreflective deposits can be present within the retinal interface (**A**); during the follow-up, these deposits become smaller, enter the inner retinal layers and fade with time until complete resolution (**B**). In the active phase, the choroid is thick and hyporeflective (**A**) and returns to normal thickness during follow-up, becoming gradually hyperreflective (**B**). The presence of an epiretinal membrane (**asterisk**) is visible over the scarred lesion (**B**).

**Figure 12:**
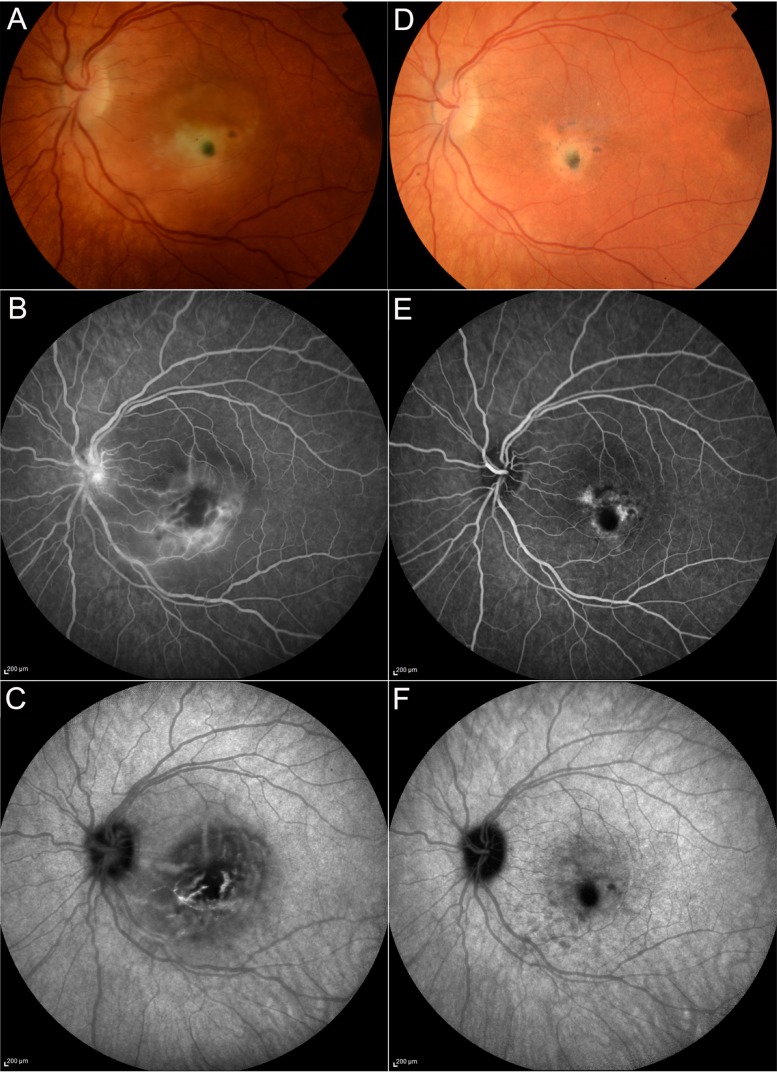
Case 4, Punctate outer retinal toxoplasmosis. Fundus examination (**A**) showing the presence of a yellowish lesion with a central grey spot on the macula, with indefinite borders, associated with perilesional edema. FA in the late phase displayed the presence of an enlarged foveal avascular zone (FAZ) corresponding to the area of detachment with perilesional leakage and papillitis (**B**). Intermediate phase ICGA evidenced a macular hypocianescent area with indefinite borders and hypercianescent striae inside the lesion (**C**). After the treatment, macular edema resolved (**D**). FA evidenced the presence of a hypofluorescent macular scar associated with RPE alterations (hyperfluorescent) in the early and late stages of the exam (**E**); a hypocianescent area is visible on ICGA (**F**).

**CASE 4**—We report a clinical case of a 32-year-old Caucasian woman who referred to sudden visual loss in her left eye. LogMAR BCVA was 1.7. The anterior segments were normal, and there were no keratic precipitates or anterior chamber reaction. OCT evidenced the presence of a severe serous retinal detachment with choroidal thickening. Photoreceptor layer was absent, and there were multiple white dots inside the detachment area (Figure 11). Fundus examination (Figure 12) showed the presence of a yellowish lesion with a central grey spot on the macula, with indistinct borders, associated with perilesional edema.

FA in the late phase displayed the presence of an enlarged foveal avascular zone (FAZ) corresponding to the area of detachment with perilesional leakage and papillitis. Intermediate phase ICGA evidenced a macular hypocianescent area with indefinite borders and hypercianescent striae inside the lesion (Figure 12).

Serological tests were performed; the patient was positive for *T. gondii* IgG and IgM. A diagnosis of punctate outer toxoplasmosis was made. Azithromycin (500 mg daily) and prednisone after the second day (1 mg pro kg, tapered slowly) was started. After a month, left eye BCVA was 1.0 LogMAR. Serous retinal detachment disappeared, and the presence of a disrupted photoreceptor layer with RPE elevation areas was observed on OCT scans along with the presence of an epiretinal membrane over the inactive lesion. FA and ICGA evidenced the presence of a macular scar (hypofluorescent; hypocianescent) associated with RPE alterations (hyperfluorescent) in the early and late phases of FA (Figure 12).

## 3. Results and Discussion

In our tertiary referral center more than 60 patients with syphilis, tuberculosis and toxoplasmosis have been evaluated in the last two years: In 60% of cases the ophthalmological evaluation was secondary to a previous diagnosis of an infectious disease, while in the remaining cases the ophthalmologist, with the help of a multimodal imaging examination and clinical evaluation, represented the physician who leads to the diagnosis. This was particularly relevant in patients affected by syphilis, in which the ophthalmological assessment allowed for a first time diagnosis in 70% of cases. To our knowledge, limited data is present in the literature about how often the ophthalmologist is the first one to make a diagnosis in described clinical entities. A recent study by Zhang et al. [70] found that in all patients evaluated for uveitis in the 85.7% of cases the diagnosis of syphilis was made by the ophthalmologist, while in the remaining cases a previous diagnosis was made.

Although laboratory testing and serology still remain the gold standard in the diagnosis of these diseases, an abnormal ocular assessment must represent a warning sign and lead to a thorough examination of the patient.

It must be said that the patients whose exams have been shown in this paper are all of Caucasian ethnicity; however, the most common and some atypical manifestations have been shown. Given that infectious pathogens-immune system interplay leads to the clinical manifestation, immunological genetic basis (including human leukocyte antigen) might be different between patients, with various subsequent patterns of presentation, and this should be taken into account during clinical evaluation.

From recent studies [71] we know that the prevalence of infectious uveitis is increasing, representing 30% of all cases in Italy; tuberculosis and syphilis accounted for 20% and almost 3% of infectious etiologies, respectively. On the other hand, the prevalence of ocular toxoplasmosis has reduced in recent years from 6.9% to 4.7%; this finding is probably due also to prenatal screening and testing. Our results confirm how in these life-threatening pathologies a prompt diagnosis is mandatory and may benefit from a multidisciplinary and multimodal imaging approach, especially during ophthalmological evaluation.

## Figures and Tables

**Table 1:** Syphilis clinical features.

Clinical Features	%
**Retinitis**	14
**Optic neuritis**	14
**Anterior uveitis (plus iris gumma)**	3.5
**Neuroretinitis**	3.5
**Intermediate uveitis**	3.5
**Retinal vasculitis**	3.5
**Posterior placoid chorioretinitis**	58

**Table 2:** Syphilis ocular complications (318 eyes).

Ocular Complications	%
**Cataract**	12.9
**Ocular Hypertension**	4.7
**Posterior Synechiae**	4.7
**Chorioretinal Scarring**	3.8
**Epiretinal Membrane**	3.8
**Macular Edema**	3.1
**Optic Disc Atrophy**	3.1
**Retinal Detachment**	2.5
**Proliferative Vitreoretinopathy**	1.6
**Phthisis Bulbi**	1.3
**Others**	2.2
**Total**	43.7

**Table 3:** Clinical features of Tuberculosis.

Clinical Features		%
**Uveitis**	Posterior uveitis	36.3
	Intermediate uveitis	15.9
	Anterior uveitis	12.5
	Panuveitis	35.3
**Vitreous haze**		45.4
**Snowballs**		16.2
**Snowbanking**		6.1
**Disc hyperemia/edema**		20.5
**Macular edema**		17.6
**Choroidal involvement**		64.4
**Retinal vasculitis** (with occlusive features)		41.4
**Retinal vasculitis** (without occlusive features)		31.5

**Table 4:** Complications and manifestations of Toxoplasmosis.

Ocular Complications	%
**Chorioretinitis**	50
**Isolated Retinal Tear**	6
**Retinal Detachment**	6
**Retinal Vascular Occlusion**	5
**Pre-Retinal Membrane**	7
**Macular Edema**	12
**Choroidal Neovascularization**	<1
**Vitreous Hemorrhage**	2
**Optic Atrophy**	4
**Cataract**	5–13
**Raised Intraocular Pressure**	30–38
**Retinochoroiditis recurrence** (at 5 years)	79

Blurry vision and scotomas are the most frequently reported symptoms.
